# MiR-4269 suppresses the tumorigenesis and development of pancreatic cancer by targeting ZEB1/OTX1 pathway

**DOI:** 10.1042/BSR20200010

**Published:** 2020-06-10

**Authors:** Xin Sui, Zhenghui Sui

**Affiliations:** Department of General Surgery, The people’s Hospital of Danyang, Affiliated Danyang Hospital of Nantong University, No. 2, Xinmin RD, Danyang 212300, Jiangsu Province, China

**Keywords:** miR-4269, OTX1, pancreatic cancer, ZEB1

## Abstract

As one of the most prevalent malignant tumors, pancreatic cancer (PC) is a leading fatal cancer worldwide. Surging evidence has unraveled that miRNAs are involved in the occurrence and progression of multiple cancers, including PC. The tumor suppressor effects of miR-4269 have been certified in gastric carcinoma. However, the potential function of miR-4269 remains largely unclear, which drives us to identify the role of miR-4269 in PC development. In the present study, we determined the expression pattern of miR-4269 in PC cells and normal cells. Results of RT-qPCR analysis illuminated that miR-4269 expression level in PC cells was lower than that in normal cells. Functional assays demonstrated that up-regulation of miR-4269 obviously inhibited the proliferation, migration and invasion of PC cells. In order to elucidate the mechanism governing miR-4269 in PC, we carried out bioinformatics analysis and further experimental investigations. Our results validated that ZEB1 was a direct target of miR-4269. Additionally, ZEB1 activated the transcription of OXT1. More importantly, miR-4269 attenuated the expression level of OXT1 via targeting ZEB1. Ultimately, our findings confirmed that miR-4269 served as a cancer suppressor in PC through regulation of ZEB1/OTX1 pathway, which suggested that miR-4269 might represent a promising target for the clinical treatment of PC.

## Introduction

Pancreatic cancer (PC) is a type of notorious carcinomas and one of the leading contributors responsible for deaths resulting from cancer throughout the world [1]. The incidence and mortality of PC are projected to rise annually and its median survival is less than six months, leading to a serious threat to public health care [2,3]. The severe lethality of PC arises from difficulties in early diagnosis, aggressive local invasion as well as early metastasis [4,5]. In past decades, great advance has been achieved in the clinical treatment of PC, but its prognosis is dismal and 5-year survival rate remains far below 5% [6]. On account of the aforementioned reasons, it is indispensable to gain a better understanding of the latent mechanism governing PC.

MicroRNAs (miRNAs) are deemed as a unique class of conserved small noncoding RNAs that are consisted of approximately 19–25 nucleotides [7]. A growing body of evidence has indicated that miRNAs function as crucial mediators in regulating the expression levels of genes via binding to complementary sequences in the 3′-untranslated regions (3′-UTR) of their target mRNAs [8]. Mounting investigations have revealed that miRNAs are involved in the genesis and evolution of multiple malignancies through affecting a wide spectrum of biological activities, such as cell proliferation, differentiation, metastasis, and apoptosis [9–11]. For instance, miR-193a suppresses breast cancer proliferation and metastasis by down-regulation of WT1 [12]. MicroRNA-21 modulates mTOR-STAT3 signaling pathway to affect the proliferation, apoptosis and differentiation of human renal cell carcinoma cells [13]. MiR-1285 restrains malignant biological behaviors of PC cells through negatively regulating YAP1 [14]. Although a recent study illustrates the tumor suppressor role of miR-4269 in gastric cancer tumorigenesis [15], explorations on the potential of miR-4269 in the development of PC are still scarce.

In the present study, we proposed to elucidate the biological function and molecular mechanism of miR-4269 in PC progression. Our results showed that miR-4269 acted as a tumor suppressor gene in PC. More importantly, miR-4269 impeded PC cell growth, migration, and invasion via targeting ZEB1/OTX1 axis. Our study provided strong evidence for the possibility of miR-4269/ZEB1/OTX1 to be a novel therapeutic approach of PC patients.

## Materials and methods

### Cell culture

Human normal pancreatic epithelial cell line HPDE and four human pancreatic cancer cell lines (PANC-1, AsPC-1, BxPC-3, and HPAC) were bought from Cell Resource Center of Shanghai Institute of Life Sciences (Shanghai, China). All the cells were cultivated in DMEM (Gibco, Carlsbad, CA, U.S.A.) with 10% FBS (Gibco), 100 μg/ml streptomycin, and 100 U/ml penicillin at 37°C in the presence of 5% CO_2_.

### Cell transfection

For enhanced expression of miR-4269, miR-4269 mimic and NC mimic obtained from Invitrogen (New York, U.S.A.) were adopted. Full length of ZEB1 was cloned into pcDNA3.1 vector purchased from Genepharma (Shanghai, China) to overexpress ZEB1 and empty vector pcDNA3.1 served as the negative control. The shRNAs against OTX1 (termed as sh-OTX1) were employed to knock down OTX1 with non-targeted shRNA (named as sh-NC) as the negative control. AsPC-1 and BxPC-3 cells were transfected with corresponding plasmids using Lipofectamin 2000 (Invitrogen) recommended by the supplier.

### RT-qPCR analysis

According to the supplier’s instructions, total RNA was isolated from PC cells with TRIzol reagent (Invitrogen). The SuperScript RT kit (Fermentas, Ottawa, Canada) was adopted to synthesize cDNA. The RT-qPCR assay was then performed on a Applied Biosystems 7500 Real-Time PCR system (Applied Biosystems, Foster City, U.S.A.) by utilizing SYBR Green PCR Master Mix (Takara Bio, Otsu, Japan). The 2^−ΔΔCT^ method was adopted to calculate gene expression levels. GAPDH and U6 were employed as internal controls for normalization. Primers were shown as follows: miR-4269: 5′-GCAGGCACAGACAGCCCTG-3′ (sense) and 5′-GAACATGTCTGCGTATCTC-3′ (antisense); ZEB1: 5′-GATGACCTGCCAACAGACCA′ (sense) and 5′-CCCCAGGATTTCTTGCCCTT-3′ (antisense); OTX1: 5′-CTGCTCTTCCTCAATCAATGG-3′ (sense) and 5′-ACCCTGACTTGTCTGTTTCC-3′ (antisense); U6: 5′-GCTTCGGCAGCACATATACTAAAAT-3′ (sense) and 5′-CGCTTCAGAATTTGCGTGTCAT-3′ (antisense).

### Cell-counting kit-8 assay

The cell-counting kit-8 (CCK-8) assay was implemented to assess cell proliferation in accordance with the manufacturer’s protocols. In briefly, AsPC-1 and BxPC-3 cells were seeded into 96-well plates at the density of 1 × 10^3^ per well and incubated at 37°C. 0, 24, 48, or 72 h later, each well was treated with 10 μl of CCK-8 and maintained at 37°C for another 2 h. The OD value at a wavelength of 450 nm was tested by a microplate reader (Bio-Tek, Winooski, U.S.A.).

### 5-Ethynyl-2′-deoxyuridine assay

Cell proliferation was also estimated with the 5-ethynyl-2′-deoxyuridine (EdU) DNA Proliferation *in vitro* Detection kit (RiboBio, China) following the supplier’s instructions. Transfected AsPC-1 and BxPC-3 cells were inoculated into 96-well plates and treated with 50 μmol/l of EdU at 37°C for 2 h. Thereafter, cells were stained with 1×Apollo reaction cocktail after fixation in 4% paraformaldehyde. DAPI (Sigma–Aldrich, St. Louis, U.S.A.) was utilized to stain cell nuclei and then EdU-positive cells were photographed with a fluorescence microscope (Nikon, Japan).

### Wound healing assay

1×10^6^ transfected cells were plated to 6-well plates and grown to 90% confluence. The scratch wound was formed with a 200 μl tip, and cells were cultured at 37°C for 48 h after washing with PBS. The wound closure was monitored and captured by a microscope at 0 and 48 h after cells were scratched.

### Transwell invasion assay

Transwell assay was conducted by using the transwell membrane coated with Matrigel (BD Biosciences, Franklin Lakes, U.S.A.). AsPC-1 and BxPC-3 cells were harvested, resuspended in 200 µl serum-free medium and seeded into the upper chamber. Six hundred microliters DMEM complemented with 10% FBS was added to the lower chamber. Twenty-four hours of incubation later, cells that went through the membrane were fixed in 4% formaldehyde, stained by 0.1% crystal violet (Beyotime, Wuhan, China) and subsequently counted in five randomly selected view fields under a microscope.

### Western blot assay

Proteins from AsPC-1 and BxPC-3 cells were extracted with RIPA buffer and the concentration of total protein was assessed by the BCA™ Protein Assay Kit (Beyotime, Beijing, China). Equal amount of proteins were fractionated by SDS-PAGE and electrophoretically transferred onto PVDF membranes. Afterwards, membranes were sealed with 5% defatted milk in TBST buffer and went through incubation with primary antibodies against ZEB1 and β-actin purchased from (Abcam, Cambridge, U.S.A.) all night at 4°C. Then, membranes were probed by secondary antibody and visualized with an enhanced chemiluminescence (ECL) detection kit (Millipore, Billerica, U.S.A.). β-Actin was employed as an endogenous control.

### RNA pull-down assay

After transfection with biotinylated miRNA-NC (named as bio-NC), biotin-labeled wild-type miR-4269 (termed as bio-miR-4269-WT) or and biotin-marked mutant miR-4269 (bio-miR-4269-Mut), AsPC-1 and BxPC cells were harvested and lysed in specific lysis buffer (Ambion, Austin, U.S.A.). Following this, cell lysates were added with M-280 streptavidin magnetic beads (Sigma–Aldrich) coated with RNase-free BSA and yeast tRNA (Sigma–Aldrich) and incubated at 4°C for 4 h. The bound RNAs were eluted from the beads, purified and subjected to RT-qPCR assay.

### Luciferase reporter assay

The fragments of wild-type ZEB1 3′-UTR containing the potential binding sites for miR-4269 were inserted into luciferase reporter vectors pMIR-GLO (Promega, Madison, U.S.A.) and referred to as ZEB1-WT. Similarly, the 3′-UTR of mutant ZEB1 was ligated into pMIR-GLO plasmids to construct ZEB1-Mut. Subsequently, cells were co-transfected with the corresponding reporter plasmids and miR-4269 mimic or NC mimic by Lipofectamine 2000 (Invitrogen). 48 h later, the relative luciferase activity was determined with the aid of the Dual-Luciferase Reporter Assay System (Promega) based on the product manuals.

### Chromatin immunoprecipitation experiment

Chromatin immunoprecipitation (ChIP) experiment was completed with an EZ-ChIP kit in line with the supplier’s instructions (Millipore). In short, AsPC-1 and BxPC cells were cross-linked by 1% formaldehyde, sonicated and then immunoprecipitated with anti-ZEB1 (Millipore) or negative control anti-IgG (Millipore). The expression level of OTX1 promoter in precipitated DNA was analyzed by RT-qPCR assay. All data were represented as relative percentage relative to the results of input DNA.

### Statistical analysis

All assays were repeated at least three times and results were expressed as the mean ± SD. GraphPad Prism 6 (Graph Pad Software, La Jolla, U.S.A.) was employed to implement statistical analyses. Differences between two groups were analyzed by Student’s *t*-test. The one-way ANOVA was adopted for comparisons among three or more groups. *P* < 0.05 was considered to indicate statistically significant.

## Results

### Overexpression of miR-4269 repressed PC cell proliferation, migration, and invasion

**Figure 1:**
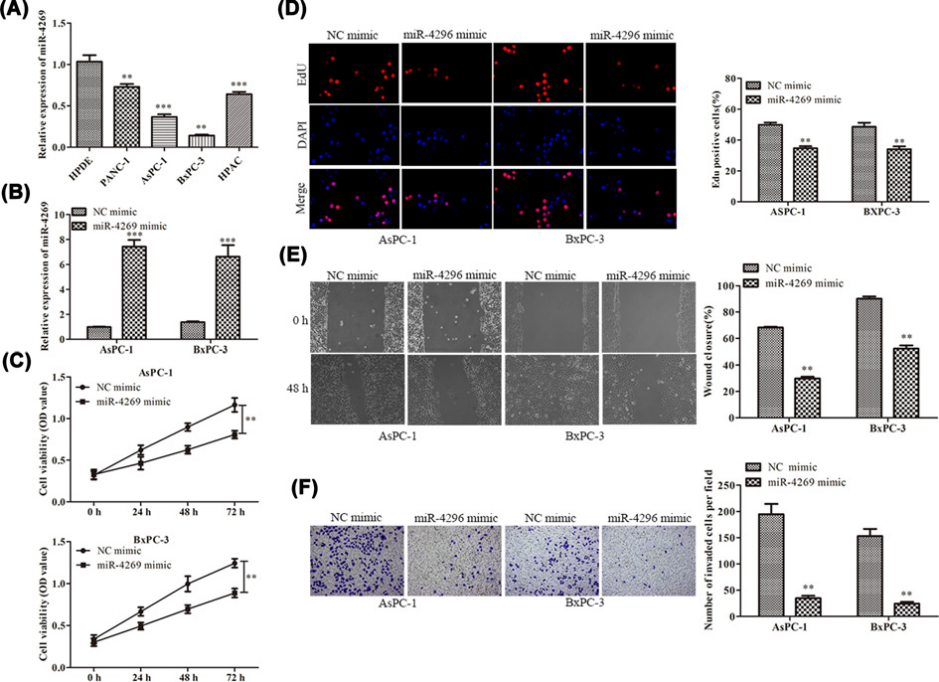
Overexpression of miR-4269 repressed PC cell proliferation, migration, and invasion (**A,B**) RT-qRCR analysis, (**C**) CCK-8 assay, (**D**) EdU assay, (**E**) wound healing assay, (**F**) transwell invasion assay were adopted to identify the functional role of miR-4269 in PC. All results were presented as mean ± SD from at least three independent assays. **P < 0.01, ***P < 0.001 versus control group.

In order to identify the expression level of miR-4269 in PC, we carried out RT-qPCR analysis and discovered that miR-4269 was weakly expressed in PC cells (PANC-1, AsPC-1, BxPC-3, and HPAC) compared with normal pancreatic epitheliums HPDE (Figure 1A). As AsPC-1 and BxPC-3 cells exhibited the lowest miR-4269 expression level, we transfected miR-4269 mimic into these indicated cells and the efficiency of miR-4269 overexpression was certified by RT-qPCR (Figure 1B). Our observations showed that up-regulation of miR-4269 caused the diminution of PC cell viability (Figure 1C). Consistently, the EdU assay indicated that miR-4269 mimic restricted cell proliferation in PC (Figure 1D). The wound healing assay disclosed that forced expression of miR-4269 led to the decreased wound closure rate of AsPC-1 and BxPC-3 cells (Figure 1E). Additionally, cell invasion was markedly suppressed owing to miR-4269 up-regulation (Figure 1F). Collectively, the aforementioned data elucidated that ectopic expression of miR-4269 inhibited the deterioration of PC via regulating cell proliferation, migration, and invasion.

### ZEB1 was a downstream target of miR-4269

**Figure 2:**
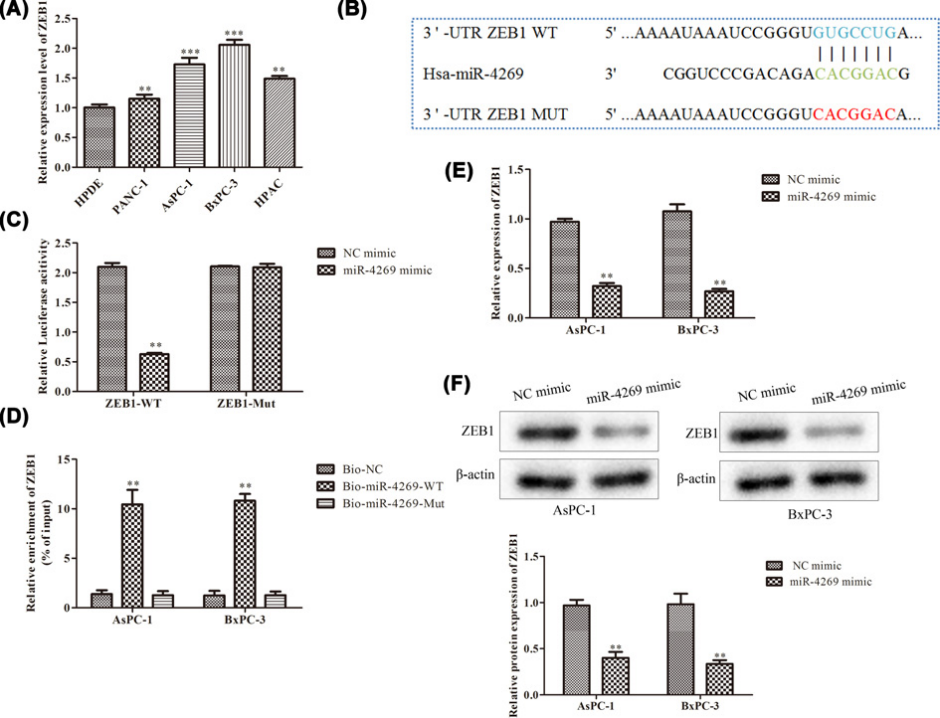
ZEB1 was a downstream target of miR-4269 (**A**) RT-qPCR. (**B**) The putative miR-4269 binding sites in 3′-UTR of ZEB1. (**C**) Luciferase reporter assay and (**D**) RNA pull down were implemented to certify the association between miR-4269 and ZEB1. (**E**) RT-qPCR and (**F**) Western blot were used to determine the effects of miR-4269 mimic on ZEB1 mRNA and protein levels. All results were presented as mean ± SD from at least three independent assays. **P < 0.01, ***P < 0.001 versus control group.

Considering that ZEB1 was a well-known oncogene in PC, we first determined the level of ZEB1 in PC cells. In contrast with normal cells, ZEB1 expression level was dramatically enhanced in PC cells (Figure 2A). By employment of bioinformatics tool starBase, it was found the putative binding sites of ZEB1 with miR-4269 (Figure 2B). As a result, we intended to verify the association between miR-4269 and ZEB1. Luciferase reporter assay illustrated that miR-4269 mimic impaired the luciferase activity of ZEB1-WT, while no obvious alteration was viewed in the mutant form of ZEB1 (Figure 2C). RNA pull-down assay revealed the overt enrichment of ZEB1 was only observed in the complex pulled downed by miR-4269, further proving that ZEB1 directly bound with miR-4269 (Figure 2D). Furthermore, enhanced expression of miR-4269 lessened the mRNA level of ZEB1 (Figure 2E). Western blot ulteriorly validated the suppressive role of miR-4269 in ZEB1 protein expression level (Figure 2F). In a word, up-regulation of miR-4269 negatively modulated ZEB1 expression level in PC.

### MiR-4269 regulated the transcription of OTX1 through suppressing ZEB1

**Figure 3:**
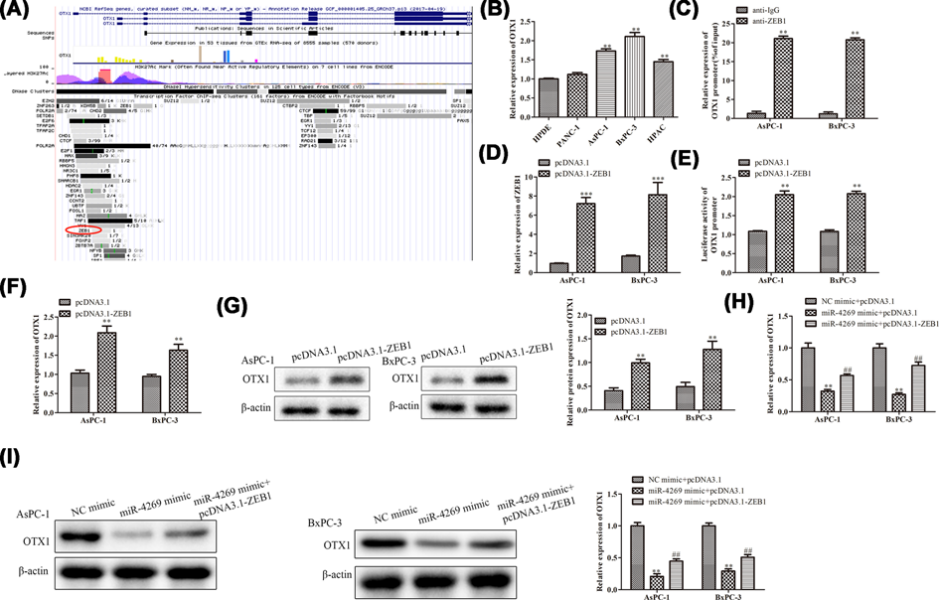
MiR-4269 regulates the transcription of OTX1 through suppressing ZEB1 (**A**) The predicted result of UCSC website. (**B**) RT-qPCR. (**C**) ChIP experiment. (**D**) RT-qPCR. (**E**) Luciferase reporter assay. (**F**) RT-qPCR and (**G**) Western blot were applied to detect the mRNA and protein expression of OTX1 when ZEB1 was overexpressed. (**H**) RT-qPCR. (**I**) Western blot. All results were presented as mean ± SD from at least three independent assays. **P < 0.01, ***P < 0.001 versus control group. ^##^*P*<0.01 versus miR-4269 mimic + pcDNA3.1 group.

Thereafter, we uncovered the potential of transcription factor ZEB1 to bind to the promoter region of OTX1 through browsing UCSC website (Figure 3A). Compared with normal cells, OTX1 expression was significantly elevated in PC cells (Figure 3B). Besides, the ChIP experiment expounded that OTX1 promoter was abundantly enriched in compound precipitated by anti-ZEB1 antibody (Figure 3C). Then, we overexpressed ZEB1 in AsPC-1 and BxPC-3 cells (Figure 3D). Results of luciferase reporter assay showed that the luciferase activity of OTX1 promoter was heightened by ectopic expression of ZEB1 (Figure 3E). In agreement with the foregoing findings, RT-qPCR assay and Western blot revealed that ZEB1 up-regulation contributed to the augment of OTX1 expression level, unveiling ZEB1 as a transcriptional activator of OTX1 (Figure 3F,G). By the reason of the targeting relationship between miR-4269 and ZEB1, we presumed that miR-4269 might regulate OTX1 via inhibiting ZEB1. Our findings delineated that overexpression of miR-4269 notably weakened OTX1 expression at both mRNA and protein levels, and therewith OTX1 mRNA and protein expression was recovered when ZEB1 was up-regulated (Figure 3H,I). Taken together, these results supported that miR-4269 suppressed OTX1 expression via targeting ZEB1.

### The role of miR-4269 in PC carcinogenesis was mediated by OTX1

**Figure 4:**
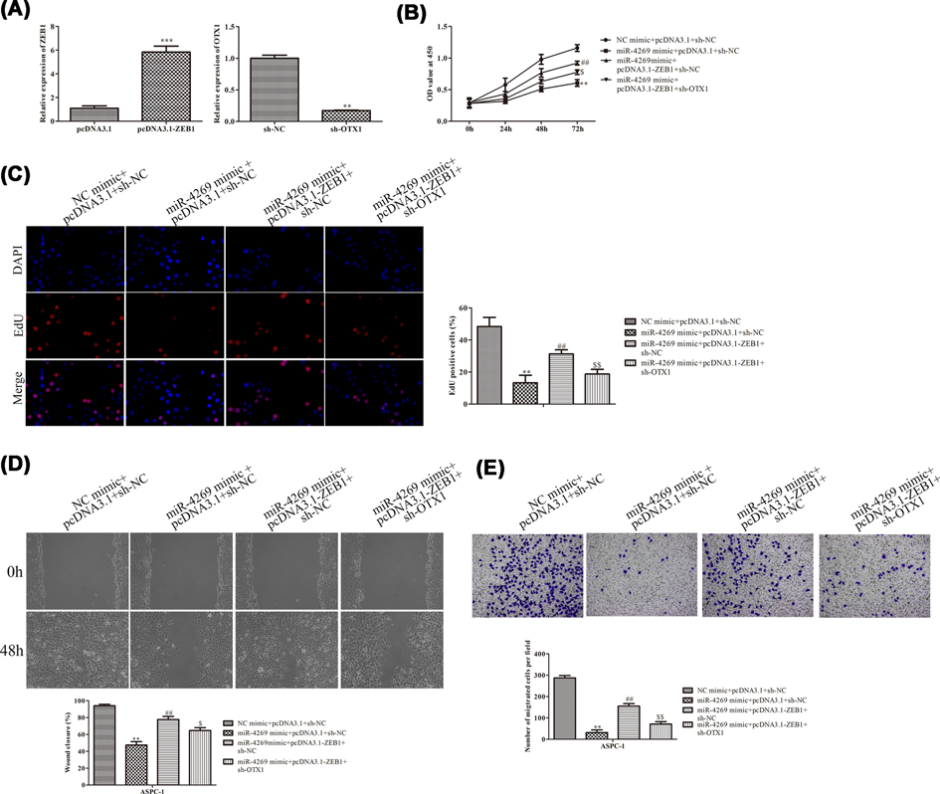
The role of miR-4269 in PC carcinogenesis was mediated by OTX1 To confirm the effects of ZEB1/OTX1 axis on PC progression caused by miR-4269. Following miR-4269 expression was enhanced, PC cells were treated with pcDNA3.1/ZEB1 with empty vector as negative control, and then transfection with sh-OTX1 or sh-NC. (**A**) RT-qPCR. (**B**) CCK-8 assay. (**C**) EdU assay. (**D**) Wound healing assay. (**E**) Transwell invasion assay. All results were presented as mean ± SD from at least three independent assays. **P < 0.01, ***P < 0.001 versus control group. ^##^*P*<0.01 versus miR-4269 mimic + pcDNA3.1 + sh-NC;^ $^*P*<0.05, ^$$^*P*<0.01 versus miR-4269 mimic + pcDNA3.1-ZEB1 + sh-NC.

Based on the above findings, we performed the rescue assays to explore whether miR-4269 exerted its oncogenic function by modulation of OTX1. After transfection, RT-qPCR analysis testified that ZEB1 level was observably elevated and OTX1 expression was knocked down in AsPC-1 cells (Figure 4A). The CCK-8 and EdU assays demonstrated that miR-4269-meidated weakened cell proliferative ability of AsPC-1 cells was promoted by enhanced expression of ZEB1 and then retrieved by silencing of OTX1 (Figure 4B,C). The wound healing assay revealed that cell migration repressed by overexpression of miR-4269 was recovered due to up-regulation of ZEB1, meanwhile the role of miR-4269/ZEB1 axis in cell migrative capacity were abrogated when OTX1 was down-regulated (Figure 4D). Concordant with these findings, we observed that ectopic expression of ZEB1 abolished the regulatory impacts of miR-4269 mimic on cell invasion and subsequently knockdown of OTX1 reversed cell invasion (Figure 4E). By the large, above results provided strong evidence that miR-4269 functioned as a tumor suppressor in PC progression via suppression of ZEB1-regulated OTX1.

## Discussion

PC is considered as a leading contributor to cancer-related deaths globally, featured by poor prognosis and rapid progression [16]. Herein, we explored the role of miR-4269 in the carcinogenesis of PC and further elucidated its molecular regulatory mechanism. Our findings indicated that miR-4269 led to the inhibition of PC cell proliferation, migration and invasion. Mechanistically, the tumor suppressive properties of miR-4269 in PC were mediated by ZEB1/OTX1 pathway.

A myriad of researches justify that miRNAs play a pivotal role in the initiation and deterioration of various human cancers, including PC [16–18]. It has been reported that abnormal expression of miRNAs is closely associated with the progression of PC [19–21]. Accumulating studies prove that miRNAs work as oncogenes or tumor-suppressor genes in PC tumorigenesis [22–24]. For instance, overexpression of miR-5100 represses the aggressive phenotypes of PC cells via targeting PODXL [25]. MicroRNA-127 is aberrantly down-regulated and serves as a tumor suppressor in PC development [26]. MicroRNA-21 enhances 5-fluorouracil resistance in human PC cells through regulation of PTEN and PDCD4 [27]. Of note, miR-4269 has been demonstrated to retard gastric carcinoma by negatively modulating TEAD1/4 [15]. Nevertheless, the function of miR-4269 in the genesis of PC remains to be investigated. In this work, we found that miR-4269 was lowly expressed in PC cells compared with normal cells. Moreover, results of gain-of-function experiments suggested that up-regulation of miR-4269 significantly impeded the growth, migration and invasion of PC cells.

Zinc-finger E-box binding homeobox 1 (ZEB1) belongs to the family of the zinc-finger-homeodomain transcription factors which are important regulators in cell growth and differentiation [28,29]. An increasing number of literatures have confirmed the oncogenic function of ZEB1 in a wide range of malignant tumors, especially in PC [29,30]. To probe the specific molecular mechanism of miR-4269 in PC, we conducted bioinformatics analysis and discovered that ZEB1 harbored the potential binding sites with miR-4269. In concert with previous studies, ZEB1 was certified to be up-regulated in PC cells. In view of that the relationship between miR-4269 and well-known oncogene ZEB1 was still unknown and high expression of ZEB1 was correlated with the poor outcomes of PC, ZEB1 was chose for in-depth study. Luciferase reporter and RNA pull-down assays validated the interaction of ZEB1 with miR-4269. Further, ZEB1 was a direct downstream target of miR-4269. Multiple lines of evidence has expounded that OTX1 acted as a cancer facilitator in numerous malignancies, such as colorectal cancer, gastric cancer, and hepatocellular carcinoma [31–33]. As a transcription factor, ZEB1 has been testified to promote the transcriptional level of diverse RNA molecules [34,35]. By employment of UCSC database, it was predicted that ZEB1 could bind with OTX1 promoter. Additionally, OTX1 was highly expressed in PC cells as expected. Subsequently, we proofed that ZEB1 was a transcription activator of OTX1 and miR-4269 alleviated the expression level of OTX1 through targeting ZEB1. Finally, rescue assays demonstrated that miR-4269 regulated OTX1 expression to restrain the malignant behaviors of PC cells in a ZEB1-independent manner.

In summary, for the first time, we identified the biological role and regulatory mechanism of miR-4269 in the carcinogenesis of PC. Our study unveiled that miR-4269 exerted its tumor inhibitory effects on PC progression by modulation of ZEB1/OTX1 axis, which offered a new insight into the clinical treatment of PC patients.

## Abbreviations

Cell-counting kit-8: CCK-8
Chromatin immunoprecipitation: ChIP
E-box binding homeobox 1: ZEB1
EdU: 5-ethynyl-2′-deoxyuridine
microRNAs: miRNAs
PC: pancreatic cancer
3′-UTR: 3′-untranslated region
